# Supplementary material for: High-Fat Diet Consumption Induces Neurobehavioral Abnormalities and Neuronal Morphological Alterations Accompanied by Excessive Microglial Activation in the Medial Prefrontal Cortex in Adolescent Mice
Source: Int J Mol Sci. 2023 May 28;24(11):9394. doi: 10.3390/ijms24119394 (PMC10253629; doi:10.3390/ijms24119394)
Supplement: Supplementary file 1 [file ijms-24-09394-s001.zip › ijms-2383812-supplementary.pdf]

Supplementary Materials

Supplementary Figures:

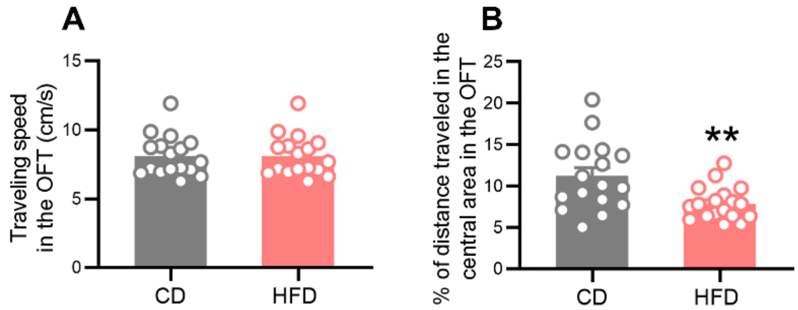

Figure S1. Effects of adolescent HFD consumption on neurobehavior in mice. Traveling speed (**A**) and proportion of distance traveled in the central area (**B**) in the OFT. Values are presented as mean ± SEM. $n = 17$–18 mice per group. ** $p < 0.01$ compared with the CD group via Mann-Whitney test. Gray and red circle points/bars represent CD- and HFD-fed mice, respectively. Each data point represents an individual mouse.

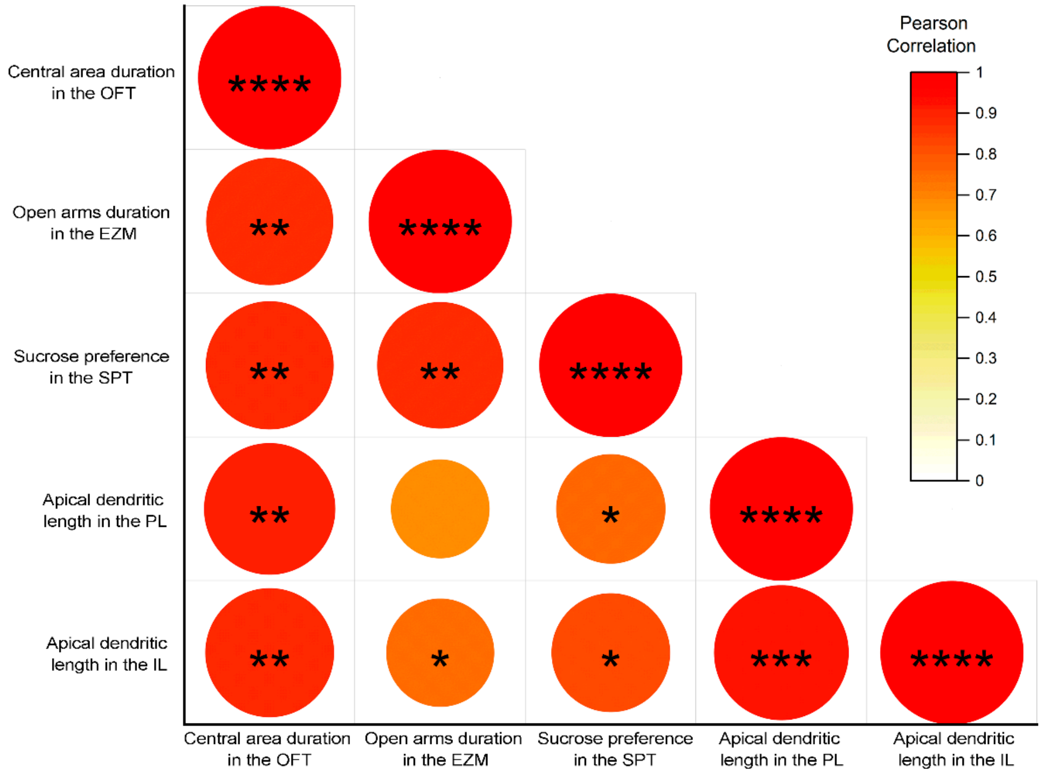

Figure S2. Pearson correlations between anxiety- and depression-like behavior and pyramidal neuronal morphology in the mPFC of the same mouse ($n = 4$ mice per group). Color intensity and size of the circle are proportional to the correlation coefficients, and asterisks indicate significance (* $p < 0.05$, ** $p < 0.01$, *** $p < 0.001$, and **** $p < 0.0001$).

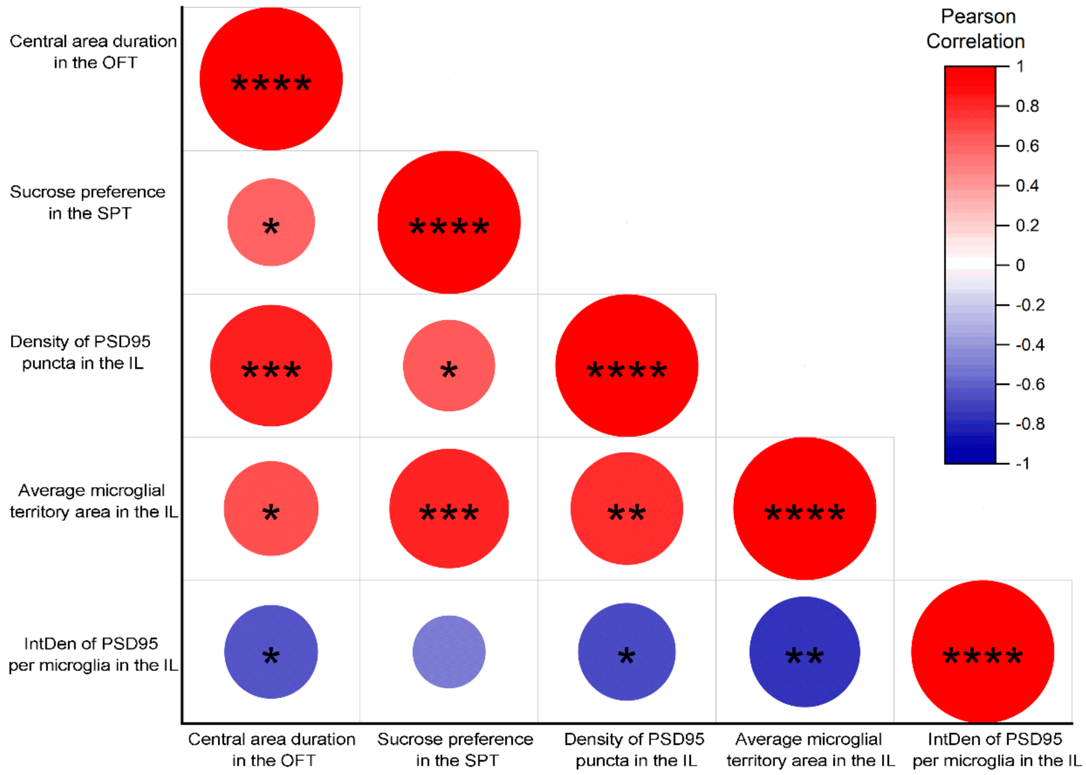

Figure S3. Pearson correlations between behavior, neuroplasticity, and microglia in the mPFC of the same mice ($n = 6$ mice per group). Positive correlations are displayed in red and negative correlations in blue color. Color intensity and the size of the circle are proportional to the correlation coefficients, and asterisks indicate significance (* $p < 0.05$, ** $p < 0.01$, *** $p < 0.001$, and **** $p < 0.0001$).
